# A local drug delivery system based on visible light-cured glycol chitosan and doxorubicin⋅hydrochloride for thyroid cancer treatment *in vitro* and *in vivo*

**DOI:** 10.1080/10717544.2018.1507058

**Published:** 2018-09-05

**Authors:** Youngbum Yoo, Sun-Jung Yoon, So Yeon Kim, Deok-Won Lee, Sewook Um, Hoon Hyun, Sung Ok Hong, Dae Hyeok Yang

**Affiliations:** a Department of Surgery, School of Medicine, The Konkuk University, Seoul, Republic of Korea;; b Department of Orthopedic Surgery, Research Institute of Clinical Medicine of Chonbuk National University, Biomedical Research Institute of Chonbuk National University Hospital, Jeonju, Republic of Korea;; c Department of Dental Hygiene College of Health Sciences, Cheongju University, Cheongju, Republic of Korea;; d Department of Oral & Maxillofacial Surgery, Kyung Hee University Dental Hospital at Gangdong Kyung Hee University, Seoul, Republic of Korea;; e Department of Veterinary Surgery, College of Veterinary Medicine, Seoul National University, Seoul, Republic of Korea;; f Department of Biomedical Sciences, Chonnam National University Medical School, Gwangju, Republic of Korea;; g Department of Dentistry, Catholic Kwandong University, School of Medicine, International St. Mary’s Hospital, Incheon, Republic of Korea;; h Institute of Cell and Tissue Engineering, College of Medicine, The Catholic University of Korea, Seoul, Republic of Korea

**Keywords:** Glycol chitosan, visible light irradiation, doxorubicin⋅hydrochloride, local drug delivery system, thyroid cancer

## Abstract

Systemic drug delivery systems (SDDSs) for thyroid cancer treatment are associated with serious side effects including nausea, anorexia, and hair loss as a result of damage to normal tissues. In this study, we investigated the feasibility of a local DDS (LDDS) based on visible light-cured glycol chitosan (GC) hydrogel and doxorubicin⋅hydrochloride (DOX⋅HCl), called GC10/DOX, on thyroid cancer treatment *in vivo*. Visible light irradiation increased the storage modulus and swelling ratio of the GC10/DOX hydrogel precursor. The release of DOX⋅HCl from GC10/DOX exhibited two unique patterns comprising an initial burst within 18 hours, followed by a controlled and sustained release thereafter. *In vitro* cell viability testing showed that GC10/DOX had a greater antitumor effect than free DOX⋅HCl and GC10 hydrogel controls. *In vivo,* local injection of GC10/DOX near tumor tissue led to a superior antitumor effect compared with controls consisting of free DOX⋅HCl intravenously injected to the tail vein of thyroid cancer-bearing mouse and GC10 hydrogel subcutaneously injected near the tumor. Altogether, our results suggest that GC10/DOX may have clinical potential for thyroid cancer treatment.

## Introduction

Thyroid cancer is the most common malignancy of the endocrine system and is the ninth most common cancer (Nguyen et al., 2015). Current modalities for treating thyroid cancer include surgery, radioiodine therapy (I-131), and chemotherapy (Nguyen et al., 2015). When used alone, these approaches often provide insufficient therapy and thus are generally used in combination with external beam radiation therapy. In addition, these clinical treatments are often associated with serious problems such as toxic side effects to normal tissues. Therefore, numerous studies have focused on how to more specifically target cancer cells, with one approach being nanomaterial-based therapeutic agents. However, nanotechnology sometimes poses several challenges, such as insufficient loading efficiency of anticancer drugs, with regards to specific elimination of tumor cells (Rocca et al., 2012; Kuen et al., 2017).

We previously demonstrated a local drug delivery system (LDDS) based on visible light-cured hydrogels for breast cancer treatment *in vivo* (Hyun et al., 2018). Specifically, we prepared hydrogels based on methacrylated glycol chitosan (MGC) hydrogel precursors and a riboflavin photoinitiator with blue visible light irradiation (430 ∼ 485 nm) (Yang et al., 2017, Yoon et al., 2017; Hyun et al., 2018) for 10 seconds, because glycol chitosan (GC), one of the water-soluble chitosan derivatives, can have an antibacterial effect by its amine group (Yoon et al., 2017). Using this system, we showed that doxorubicin⋅hydrochloride (DOX⋅HCl), an anticancer drug used for breast cancer treatment, can be directly delivered to tumor tissue in a controlled and sustained manner. Thus, we have found that hydrogel-based drug delivery systems (DDSs) are a useful tool for effective treatment of breast cancer *in vivo* (Hyun et al., 2018).

Doxorubicin (DOX) is one of the most clinically used anticancer drugs for thyroid cancer treatment (Matuszczyk et al., 2008); however, its poor water solubility results in low bioavailabilility (Ke et al., 2008). So, in the present study, we investigated the ability of visible light-cured GC hydrogel system containing water-soluble doxorubicin⋅hydrochloride (DOX⋅HCl) to treat thyroid cancer using a mouse xenograft model. Specifically, we determined the time-dependent release behavior of DOX⋅HCl in the hydrogel system. In addition, we studied the *in vitro* and *in vivo* anticancer effects of the hydrogel system at predetermined time intervals.

## Experimental section

### Materials

Glycol chitosan (GC, ≥60% calculated by titration, crystalline, Mw ≅ 585,000 g/mol) and glycidyl methacrylate (GM) used for the preparation of the hydrogel precursor solution were supplied by Sigma-Aldrich (St. Louis, MO). Doxorubicin⋅hydrochloride (DOX⋅HCl, Tokyo Chemical Industry Co., Ltd, Tokyo, Japan) was used for thyroid cancer treatment. Cellulose membrane used for purification was purchased from Spectrum Laboratories Inc. (Rancho Dominguez, CA). The FTC-133 human follicular thyroid carcinoma cell line was obtained from American Type Culture Collection (ATCC, Manassas, VA). All chemicals were used as received without further purification.

### Preparation and characterization of visible light-cured GC hydrogel containing DOX⋅HCl

**Figure 1.**
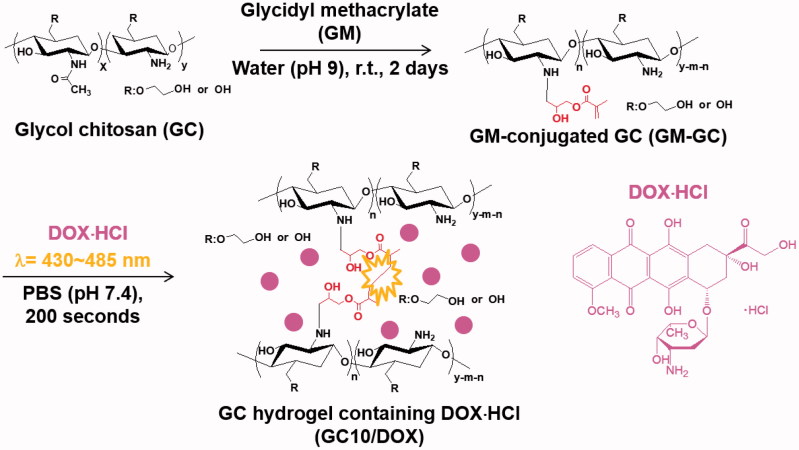
Scheme for the preparation of GC10/DOX.

Photocured GC hydrogel based on 1 *w/v*% GM-GC and 12 μM were prepared as reported in our previous studies, because the specific conditions can lead to controlled release of biomolecules in a sustained manner (Yang et al., 2017; Yoon et al., 2017; Hyun et al., 2018). Figure 1 shows the preparation route of visible light-cured GC hydrogel containing DOX⋅HCl (GC10/DOX). Briefly, to a solution of GC (0.003 mmol, 1.5 g) in water, the pH was adjusted to 9 and GM (0.05 mmol, 7 mg) was added. After epoxy ring opening reaction at room temperature for 2 days, the reactant was neutralized and dialyzed (cut-off: 20 kDa) in water for 7 days. After lyophilization at -90 °C for 7 days, the resulting white solid (GM-GC; 1 *w/v*%) was dissolved in water and riboflavin (12 μM) was added. The mixture was then homogenously dispersed with continuous stirring. After adding DOX⋅HCl (2 mg/mL), the mixture was irradiated with blue visible light (430–485 nm, 2100 mW/cm^2^, light-emitting diode (LED) curing light, Foshan Keyuan Medical Equipment Co., Ltd., Guangdong, China) for 10 seconds for hydrogelation.

An AR 2000 EX rheometer (TA instruments, New Castle, DE) was employed for examining the storage and loss moduli of GC10/DOX hydrogel precursor before and after blue visible light irradiation. The hydrogel precursor was placed on a metal mount set with a cone and plate geometry with a 4 cm diameter and 1° cone angle. The sample was monitored as a function of frequency from 0 Hz to 100 Hz.

The swelling ratio was calculated as the ratio of the swollen weight to the initial weight of the photo-cured hydrogel. At predetermined time intervals, hydrogel immersed in water was extracted, washed three times with water, and weighed.

### *In vitro* release of DOX⋅HCl

The release of DOX⋅HCl from GC10/DOX was examined as described previously (Hyun et al., 2018). Briefly, DOX⋅HCl (3.5 μmol, 2 mg), GM-GC (1 *w/v* %), and riboflavin (12  μM) were dissolved in PBS (pH 7.4, 1 mL) and irradiated with blue visible light for 10 seconds. The resulting hydrogel was transferred to a cellulose membrane (MWCO: 3500 g/mol). After immersing in a conical tube filled with PBS (pH 7.4, 8 mL), the tube was incubated at 37 °C at 100 rpm. At predetermined time intervals (1, 3, 6, 12, and 24 h, and 2, 3, 4, 5, 6, and 7 days), effluent (2 mL) was extracted and an equal volume of fresh PBS was added. The extracted effluents were then analyzed at 480 nm using a microplate reader.

### Field emission scanning electron microscopy (FE-SEM)

Freeze-dried hydrogel samples were broken by grabbing their both sides with two tweezers and then platinum-coated using an ion sputter-coater (Eiko IB-3, Eiko Engineering Co. Ltd, Japan). FE-SEM (Inspect F; FEI, Hillsboro, OR, USA) was employed to observe the surface and cross-sectional morphologies of the samples, which performed at 30.0 kV with a magnification of 500×.

### *In vitro* cell viability test

CCK-8 reagent was used to evaluate the viability of the FTC-133 cell line *in vitro* during culture on GC10/DOX hydrogel compared to GC10 and control. Cells (5 × 10^3^ cells/well) were seeded on samples and incubated at 37 °C for 1, 3, and 7 days. At predetermined time intervals, the samples were washed with PBS and CCK-8 was added. The samples were incubated for an additional 4 hours and the optical density of supernatants was measured at 450 nm using a microplate reader.

### FTC-133 xenograft mouse model

All experimental animal protocols were approved by the institutional Animal Care and Use Committee (IACUC) of Kyung Hee University Hospital at Gangdong. For xenografts (Li et al., 2017), FTC-133 cells (5 × 10^6^ cells) were suspended in water and injected (100 μL; JEIL PHARMACEUTICAL Co. Ltd., Daegu, Korea) subcutaneously into the backs of adult (6-week-old) male BALB/c nude mice (*n* = 35, 22–25 g, D.Y. Biotech., Seoul, Korea). Upon reaching a tumor volume of approximately 250–330 mm^3^, free DOX⋅HCl was intravenously injected via the tail vein, while injectable GC based hydrogels (GC10 and GC10/DOX) were administrated locally near tumor tissue. The concentration of DOX⋅HCl was adjusted to 6 mg/kg in 100 μL of water for injections.

### *In vivo* evaluation of antitumor effect

Mice bearing tumors were divided into groups according to treatment as follows: control (untreated), free DOX⋅HCl, GC10, and GC10/DOX. Free DOX⋅HCl was intravenously injected through the tail vein of tumor-bearing mice, while the two hydrogel systems were subcutaneously injected near the tumor tissue. At predetermined time intervals, tumor volumes were calculated by the following formula: V = 0.5 × longest diameter × (shortest diameter)^2^.

### Histological evaluation

Tumour tissues resected at day 7 after sample treatment were stained with hematoxylin and eosin (H&E). The tissues were fixed in a 10% neutral formaldehyde solution overnight and then embedded in paraffin. Sample blocks were sectioned at a thickness of 4 μm, and slides were visualized by fluorescence microscopy (AX 70, TR-62A02, Olympus, Tokyo, Japan).

### Statistical analysis

All quantitative data are expressed as the mean ± standard deviation. Statistical analyses were performed with one-way analysis of variance (ANOVA) using SPSS software (SPSS Inc., Chicago, IL, USA). A value of *p < .05 was considered statistically significant.

## Results

### Storage/loss moduli of GC10/DOX

**Figure 2.**
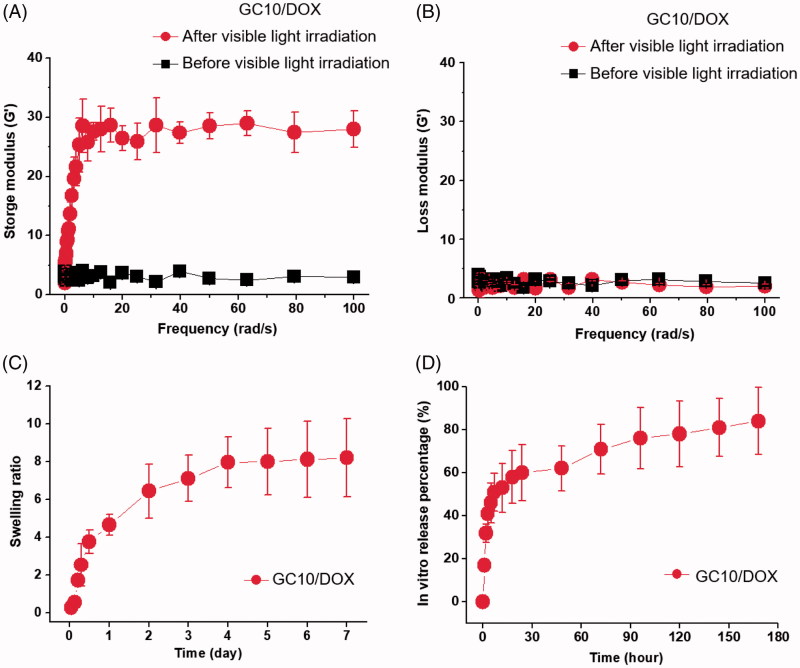
(A) Storage and (B) loss moduli of GC10/DOX hydrogel precursor before and after visible light irradiation, (C) the swelling ratio of GC10/DOX cured with the light for 10 seconds, and (D) *in vitro* release percentage of DOX as a function of time. These experiments were carried out three times.

In order to prepare the hydrogel precursor solution for visible light-cured hydrogel systems, GM-conjugated glycol chitosan (MGC) was prepared as in our previous reports (Yang et al., 2017, Yoon et al., 2017; Hyun et al., 2018). Figure 2(A and B) shows the storage and loss moduli of GC10/DOX as a function of frequency before and after visible light irradiation. There was a marginal difference between storage and loss moduli in the hydrogel precursor before light irradiation. After light irradiation for 10 seconds, the storage modulus of GC10/DOX was higher than the loss modulus, indicating that elasticity was increased as a result of the formation of a three-dimensional network among MGC chains. These changes contributed to the formation of the GC10/DOX hydrogel after visible light irradiation for 10 seconds.

### Swelling ratio of GC10/DOX

Figure 2(C) shows the swelling ratio of GC10/DOX hydrogel examined at 37 °C for 7 days. The hydrogel reached a swelling equilibrium within 5 hours with rapid swelling behavior because MGC chains loosely interconnected by visible light irradiation for 10 seconds. The swelling ratio at 5 hours was 8-fold higher than the initial state.

### *In vitro* release behavior of DOX⋅HCl

Figure 2(D) shows the *in vitro* release behavior of DOX⋅HCl in GC10/DOX at 37 °C for 7 days. The hydrogel exhibited an initial burst of DOX⋅HCl within 18 hours, after which the drug was released in a sustained manner for 7 days. These results were attributed to drug diffusion from the swollen matrix.

### Surface and cross-sectional morphologies

**Figure 3.**
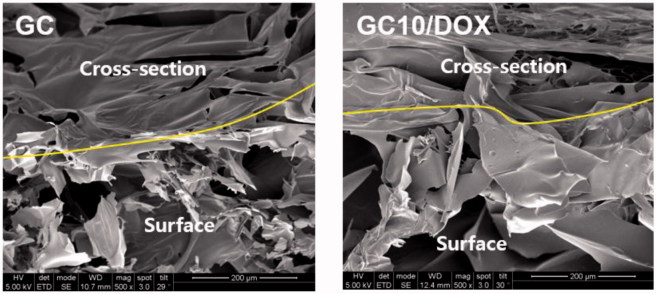
FE-SEM images of freeze-dried GC and GC10/DOX observed at 500 ×.

Figure 3 shows the surface and cross-sectional morphologies of freeze-dried GC and GC10/DOX hydrogels. They had irregular porous structure with interconnectivity irrespective of the addition of DOX⋅HCl.

### *In vitro* cell viability

**Figure 4.**
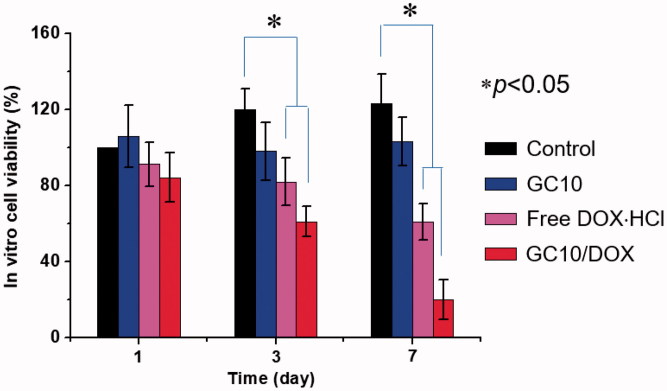
*In vitro* viability of FTC-133 cells cultured on control, GC10, free DOX⋅HCl, and GC10/DOX. The cell viability rate (%) was determined by CCK-8 assay at 1, 3, and 7 days. Error bars represent mean ± SD (*n* = 3); these experiments were repeated three times.

The viability of the FTC-133 human follicular thyroid carcinoma cell line cultured on GC10/DOX for 7 days was examined to confirm its antitumor effect *in vitro* and compared with those of control, free DOX⋅HCl, and GC10 (Figure 4). Compared with control, GC10 treated cells exhibited almost constant viability throughout the culture period and no cytotoxicity. Treatment of cells with either free DOX⋅HCl or GC10/DOX resulted in a gradual time-dependent decrease in cell viability. On day 7 of treatment, the abundance of dead cells in control, free DOX⋅HCl, GC10, and GC10/DOX treated groups was 0%, 0%, 39%, and 80%, respectively, which was attributed to the apoptosis inducted by DOX.

### *In vivo* antitumor effect

**Figure 5.**
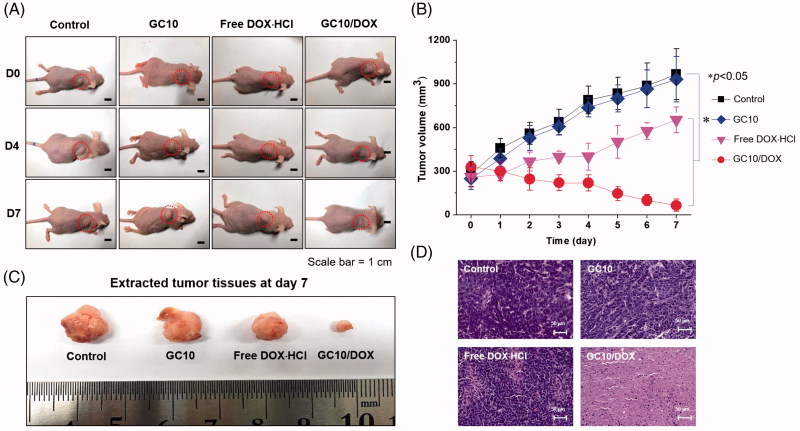
(A) Macroscopic appearances of tumors in control, GC10, free DOX⋅HCl and GC10/DOX-treated mice on day 0, 4, and 7. (B) Tumor volume (mm^3^) of tumors extracted from the samples. (C) Comparison of tumor size extracted from the samples at day 7. (D) H&E stained images of dissected tumors extracted from the samples at day 7. Error bars represent mean ± SD (*n* = 3); these experiments were repeated three times. The black and white scale bars indicate 1 cm and 50 μm, respectively.

Figure 5 shows the *in vivo* antitumor effect of GC10/DOX in FTC-133 tumor-bearing mice compared with control, free DOX⋅HCl, and GC10 treatment after 7 days. The two hydrogels were subcutaneously injected near tumor only once on the first day. On the other hand, free DOX⋅HCl was injected through the tail vein of mice only once on the first day. On gross appearance as shown in Figure 5(A), control, free DOX⋅HCl, and GC10-treated samples exhibited gradual increases in tumor volumes, while a gradual decrease in tumor volume was observed in the GC10/DOX-treated sample. To quantitatively examine changes in tumor growth, the tumor volume of each sample at predetermined time intervals was calculated (Figure 5(B)). On day 7, control, free DOX⋅HCl, and GC10-treated samples had 3.5, 3.7, and 2.5-fold larger tumor volumes relative to day 0, respectively. Conversely, the tumor volumes of mice treated with GC10/DOX on day 7 were 5.1-fold smaller than at day 0. At the same time, these results indicate that local injection of GC10/DOX is a useful tool for thyroid cancer treatment. Figure 5(C) shows the tumor tissues extracted from the sample-treated mice at day 7. Likewise, the tumor size in G10/DOX treated mouse was the smallest among the others. Figure 5(D) shows representative H&E stained images of tumors isolated from the four treatment groups. A dense distribution of cancer cells was observed in both the control and GC10 groups, supporting the cytocompatibility of GC10. The sectioned tumor tissues obtained from mice treated with DOX⋅HCl had a partial necrotic area together with the distributed cancer cells. Among GC10-based samples, local treatment with GC10/DOX resulted in significant necrosis of tumor tissue.

### Histological evaluation of tumors treated with GC10, free DOX⋅HCl, and GC10/DOX

**Figure 6.**
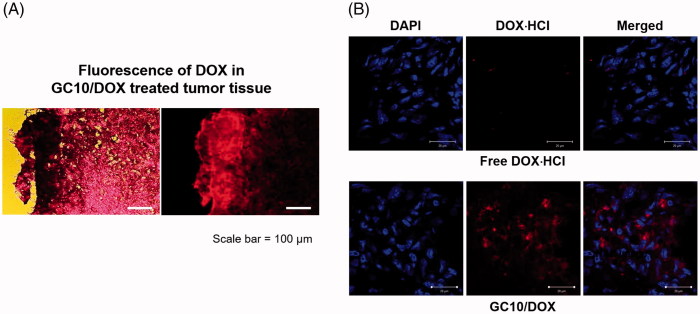
(A) Fluorescence images of DOX in dissected tumors extracted from free DOX⋅HCl and GC10/DOX treated mice and (B) their H&E stained images. The white scale bar indicates 20 μm.

To further investigate the antitumor effects of GC10/DOX *in vivo*, sectioned tumor tissues were stained with H&E and cellular behavior and tissue changes of the samples were observed as compared with control, free DOX⋅HCl, and GC10-treated tumor tissues (Figure 6(A)). In control treated mice, viable cancer cells were densely distributed throughout the tissue. Free DOX⋅HCl and GC10-treated samples exhibited a partial distribution of necrotic tissue along with a similarly dense distribution of cancer cells. However, a necrotic area was thoroughly observed in GC10/DOX-treated samples. In order to more definitively attribute DOX⋅HCl treatment to the antitumor effect, sectioned slides of free DOX⋅HCl- and GC10/DOX-treated samples were observed by fluorescence spectroscopy (Figure 6(B)). The results showed that GC10/DOX-treated samples had a greater amount of DOX⋅HCl than free DOX⋅HCl-treated samples, suggesting that the LDDS for DOX⋅HCl may be a superior tool for thyroid cancer treatment compared to systemic DDS.

### Systemic toxicity of GC10, free DOX⋅HCl, and GC10/DOX

**Figure 7.**
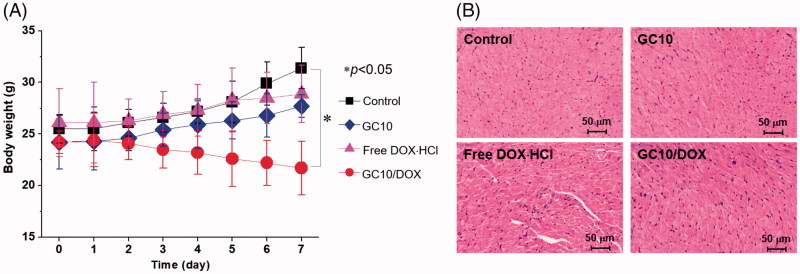
(A) Change in the body weight of control, GC10, free DOX⋅HCl and GC10/DOX-treated mice for 7 days. Error bars represent mean ± SD (*n* = 3); these experiments were repeated three times. (B) H&E stained images of dissected hearts extracted from control, GC10, free DOX⋅HCl and GC10/DOX-treated mice.

Systemic toxicity was evaluated by tracking the body weights of control, GC10-, free DOX⋅HCl-, and GC10/DOX-treated samples (Figure 7(A)). Compared with control, slight increases in body weight were observed in GC10- and free DOX⋅HCl-treated animals. In free DOX⋅HCl-treated animals, the change in body weight was attributed to limited effect of DOX⋅HCl because the drug itself is decomposed or eliminated by the opsonization in blood stream. On the other hand, in case of GC10/DOX-treated sample, the lack of cytotoxicity of GC10 and specific delivery of DOX⋅HCl were affirmed by our observation of increased body weights. To further examine the potential cardiotoxicity of free DOX⋅HCl, H&E-stained slides of heart tissues from GC10-, free DOX⋅HCl-, and GC10/DOX-treated animals were evaluated (Figure 7(B)). Whereas H&E-stained images of normal heart tissue showed no significant toxicity-related changes, intravenous injection of free DOX⋅HCl induced several abnormal cardiotoxic effects including appearance of inflammatory cells, disorganization of myocardium, and increased cytoplasmic vacuolization and myofibrillar fragmentation. H&E-stained images of heart tissues in GC10- and GC10/DOX-treated samples were similar to those of normal heart tissue, although alteration in the morphology of cardiomyocytes, albeit with decreased cytoplasmic vacuolization and myofibrillar fragmentation, was noted for animals treated with either of the GC10-based matrices. These results suggest that local injection of GC10/DOX may be superior to DDS for thyroid cancer treatment due to the presence of free DOX⋅HCl.

## Discussion

Despite significant advances in thyroid cancer treatment, locoregional recurrence occurs frequently in both early and intermediate stage disease (Grant, 2015). Therefore, there continues to be a need for novel methods for improving the cancer treatment. Localized chemotherapy delivered to tumor has several potential benefits with respect to effective cancer treatment and aims to improve antitumor effects while reducing morbidity (De Souza et al., 2010). Indeed, localized chemotherapy can diminish the toxicity of normal tissues and deliver anticancer drugs to tumor tissues in a sustained manner by avoiding systemic circulation of anticancer drugs and improving therapeutic effects (De Souza et al., 2010).

So far, several different matrices for localized chemotherapy have been described, among which thermo-responsive hydrogels have been the primary target of investigation due to their sol-gel transition behaviors at body temperature (He et al., 2017; Qi et al., 2018). However, these systems are physically cross-linked, which may make it difficult to control drug release in a desired pattern, because modulating the pore size in the matrices depends on the polymer concentration (Bhattarai et al. 2010; Parhi et al. 2017). In comparison with the physical systems, chemically cross-linked hydrogels can control drug release in modulated pore size (Parhi et al., 2017). Among chemically cross-linked matrices, photocurable hydrogel systems may easily control drug release by fine-tuning crosslinking density (Hyun et al., 2018).

We previously showed that visible light-cured GC hydrogel systems are useful LDDSs for breast cancer treatment (Figure 1), because light irradiation is cytocompatible and can be used to prepare injectable hydrogels without inducing physical damage (Hyun et al., 2018). Among various natural or synthetic polymers, we specially investigated GC because the polymer is water-soluble, biocompatible, and biodegradable (Yang et al., 2017, Yoon et al., 2007; Hyun et al., 2018). In addition, the hydroxyl or amine groups of GC can conjugate with functional groups for photo-curing (Yang et al., 2017; Yoon et al., 2017; Hyun et al., 2018).

As a continuation of our previous work, we investigated the feasibility of GC10/DOX for thyroid cancer treatment *in vitro* and *in vivo*. The storage/loss moduli and swelling ratio were examined and were in good agreement with our previous results (Hyun et al., 2018) (Figure 2(A–C)). After visible light irradiation, the polymer chains in GC10/DOX hydrogel precursor formed a 3-D network due to their interconnectivity via the radical reaction of the riboflavin photoinitiator (Hu et al., 2012). The resulting structure exhibited increased elasticity and porosity of the surface and bulk parts, respectively. Altogether, these physical and structural properties affected release behavior of DOX⋅HCl.

We, in our previous study, described how photo-cured hydrogel systems have two release patterns comprising an initial burst release, followed by a sustained controlled release thereafter (Hyun et al., 2018). These phases with anticancer drugs in aqueous solution are influenced by water penetration into the porous polymer network, swelling of hydrated polymer, and drug diffusion from the swollen network. Therefore, the release pattern of drugs in a polymer matrix is strongly related to position: drugs near the matrix surface are released during the initial burst, while those present in the bulk material undergo controlled release (Sriamornsak et al., 2007), consistent with the pattern of release shown in the present study (Figures 2(D) and 3).

DOX is one of the most well-known anticancer drugs used to treat thyroid cancer (Matuszczyk et al., 2008). Several mechanisms for the anticancer effects of DOX in the treatment of thyroid cancer have been proposed, of which inhibition of DNA synthesis by poisoning topoisomerase II (TOP2A) and intercalation with DNA are well described (Pommier et al., 2010; Agudelo et al., 2014; Yang et al., 2014). Unfortunately, DOX is poorly soluble in water and has insufficient delivery to tumor, limiting its clinical usefulness (Ke et al., 2008). In this regard, visible light-cured GC hydrogels containing DOX⋅HCl can overcome the limitations, because the HCl salt form of DOX is water-soluble and DOX⋅HCl loaded GC hydrogels can specifically deliver drug towards the tumor when injected nearby.

As reported previously (Hyun et al., 2018), pore structure is essential for loading and release of anticancer drugs, and that visible light-cured GC hydrogels are amenable to pore structure control through systemic modulation of crosslinking density. Consistent with these properties, GC10/DOX exhibited a superior antitumor effect on thyroid cancer cells *in vitro* and *in vivo*. In addition, analysis of H&E-stained tissue specimens supported the antitumor effect of GC10/DOX. Lastly, local treatment of GC10/DOX appeared to induce apoptosis of cancer cells, resulting in necrosis of tumor tissues.

**Figure 8.**
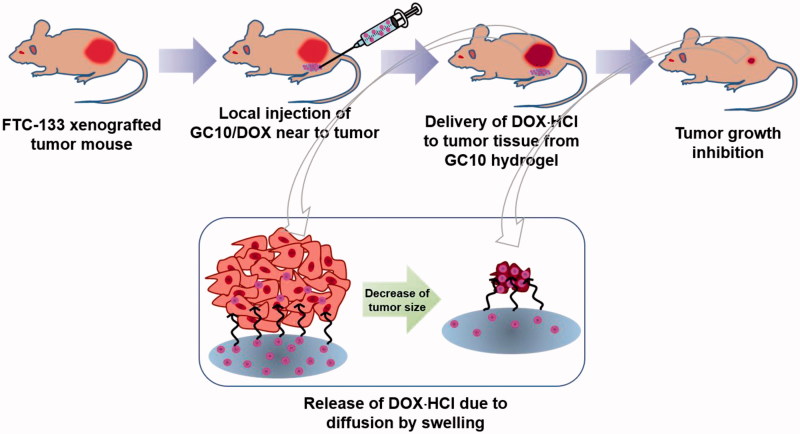
Schematic illustration showing possible mechanism of GC10/DOX on the improvement of thyroid cancer treatment. GC10/DOX was locally injected next to tumor.

Evaluation of the systemic side effects of DDSs is a prerequisite for confirming its biocompatibility *in vivo*. Many cancer patients suffer from the side effects of commercially available anticancer chemotherapy drugs. In addition, the toxicity of anticancer drug carriers is an important consideration when developing effective cancer treatments. GC10/DOX exhibited excellent biocompatibility (Figures 4–7), suggesting that the hydrogel injected near tumor tissue can result in effective drug delivery with minimal off-target tissue-toxicity along with minimized cardiotoxicity of DOX (Figure 8).

## Conclusions

In this study, we investigated the feasibility of using DOX⋅HCl loaded visible light-cured hydrogel systems on thyroid cancer treatment *in vitro* and *in vivo*. The drug-loaded hydrogel systems exhibited a porous structure that affected the release behavior of DOX⋅HCl. In addition, controlled release of DOX⋅HCl in injectable GC hydrogels located near tumor tissue resulted in a superior antitumor effect on thyroid cancer *in vivo*. Accordingly, we suggest that the injectable hydrogel system described in this study may have clinical potential for the treatment of thyroid cancer.
